# PARP1-Erk synergism in proliferating cells

**DOI:** 10.18632/oncotarget.25633

**Published:** 2018-06-26

**Authors:** Leonid Visochek, Malka Cohen-Armon

**Affiliations:** ^1^ Department of Physiology and Pharmacology, Sackler School of Medicine, Tel-Aviv University, Tel Aviv 69978, Israel; ^2^ Sagol School of Neuroscience, Tel-Aviv University, Tel Aviv 69978, Israel

**Keywords:** PARP1-Erk binding, PARP1-dependent Erk activity, c-Fos, Mouse embryonic fibroblasts

## Abstract

A synergism between PARP1 and phosphorylated Erk mediating IEG (immediate early gene) expression has been recently reported in cerebral neurons and cardiomyocytes. Stimulation induced PARP-Erk synergism was required for IEG expression underlying synaptic plasticity and long-term memory acquisition during learning. It was similarly required for cardiomyocytes development. Here, we identified this mechanism in Erk-induced gene expression promoting proliferation. This mechanism can be targeted in malignant cells.

## INTRODUCTION

Numerous signal transduction mechanisms target the MAP (mitogen activated protein) kinase phosphorylation cascade in a variety of cell types [1, 2]. In the absence of NLS (nuclear localization signal), phosphorylated Erks shuttle between the cytoplasm and the nucleus, with tendency to translocate to the nucleus in response to stimulation [3]. A possible binding of phosphorylated Erks to any nuclear protein would retain their activity in the nucleus [3, 4]. On the basis of recent findings, we suggest that the most abundant nuclear protein PARP1 acts as an anchoring protein of phosphorylated Erk2 [5–7]. Docking sites of phosphorylated Erk were identified in PARP1 domains, which are not involved in the binding of PARP1 to DNA single strand breaks [7]. Moreover, phosphorylated Erk2 induced PARP1 activation and polyADP-ribosylation in cell-free systems and in cerebral neurons and cardiomyocytes [5–7]. PARP1 bound to phosphorylated Erk2 was highly polyADP-ribosylated in the presence of very low NAD concentrations due to intramolecular modifications enhancing its affinity for NAD [5, 6]. Activated PARP1 polyADP-ribosylate its prominent substrate in the chromatin, linker histone H1 [5–7]. PolyADP-ribosylated H1 repulsion from the negatively charged DNA [7, 8] caused a local chromatin relaxation, exposing transcription factors to phosphorylation by the PARP-bound phosphorylated Erk, as indicated by chromatin immunoprecipitation (ChIP assay) technique in electrically stimulated cultured cortical neurons [7]. Phosphorylated transcription factors Elk1 and CREB induced histone H4 acetylation and IEG expression implicated in synaptic plasticity [5, 6, 9, 10]. This mechanism may explain the role of H1 polyADP-ribosylation in PARP1 dependent memory acquisition [11–13], as well as the lack of long-term memory acquisition in PARP1-KO mice [7].

Here, we identified the same mechanism active in proliferating mouse embryonic fibroblasts (MEF).

## RESULTS AND DISCUSSION

In proliferating cells, Erk-induced c-fos expression may activate transcription factor AP1 (combined of phosphorylated c-Fos protein bound to c-Jun). AP-1 promotes cyclinD expression, and cdk1 activation implicated in the initiation of mitosis [14–17]. Here we found that c-fos expression was governed by PARP1-Erk binding and synergism in stimulated MEF.

In proliferating cells, stimulation-induced PKC phosphorylation mediates proliferation via a signal transduction implicating PKC binding to diacylglycerol phosphate (DAG) in the cell membrane [14, 15]. In the depicted experiments, PKC binding to DAG was mimicked by its binding to phorbol esther (PMA). The resulting PKC phosphorylation mediates proliferation via downstream phosphorylation of Erk [14, 15].

**Figure 1:**
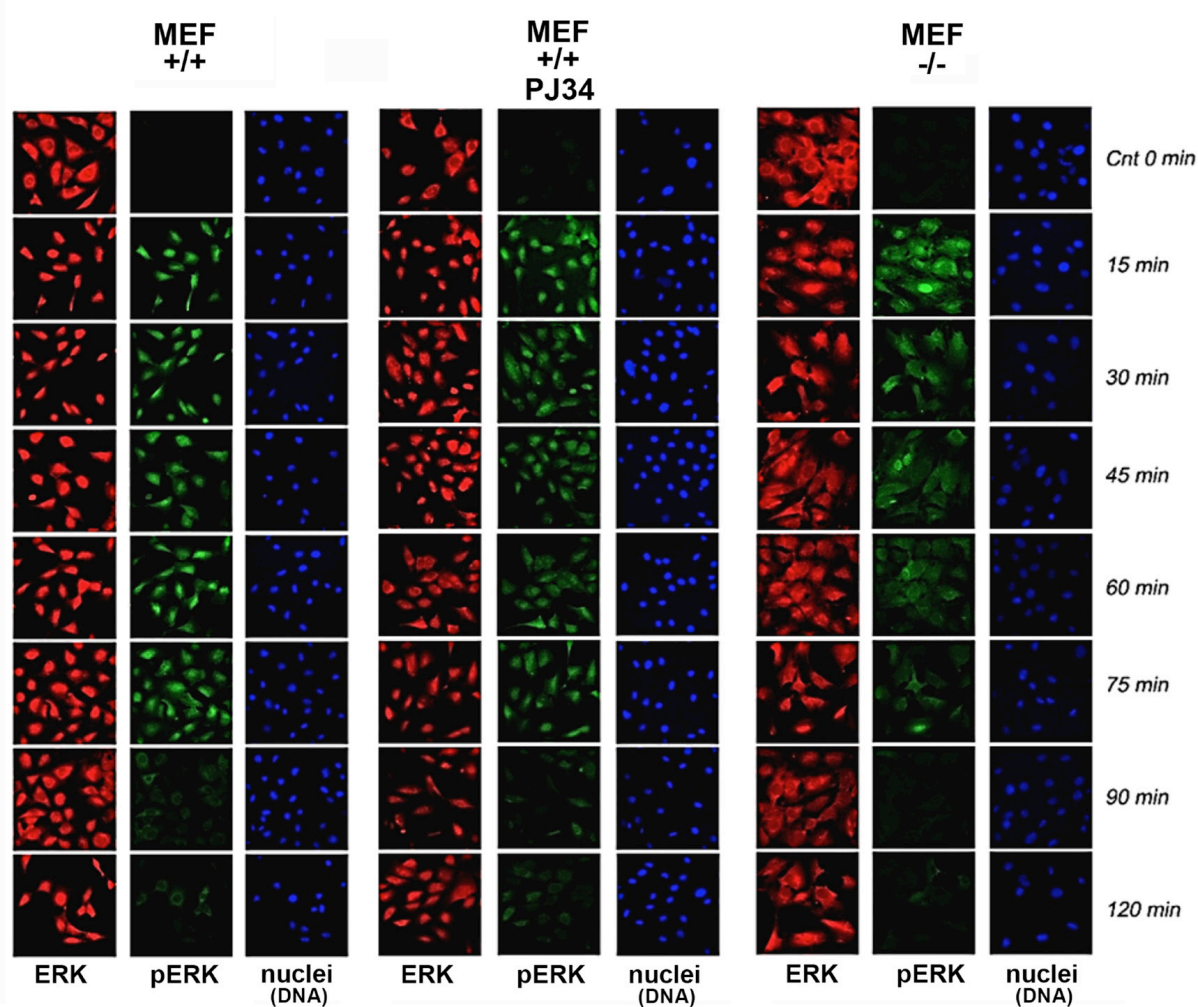
Stimulation–induced Erk phosphorylation down-regulated in nuclei of PARP1-KO MEF Immunolabeled Erk (red) and phosphorylated Erk (green) monitored by confocal microscopy during 120 min after stimulation with PMA (200 ng, 15 min) in nuclei of MEF, without and after PARP1 inhibition, and in nuclei of PARP-KO MEF. DNA is labeled with DAPI (blue).

**Figure 2:**
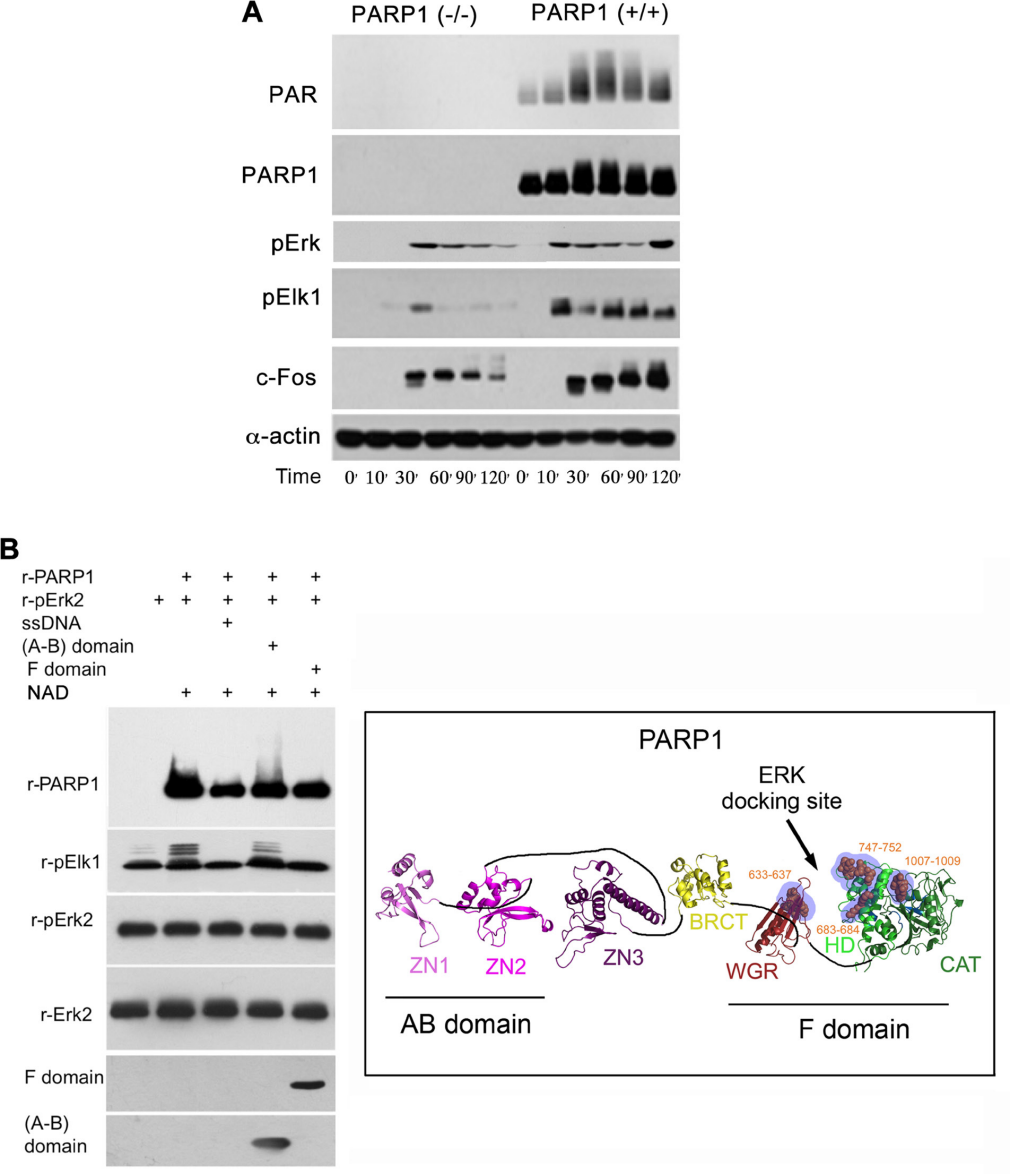
A PARP1 dependent Erk-activity in the nucleus (**A**) Immunolabeled phosphorylated Erk1/2, and transcription factors Elk1 and c-Fos monitored during 120 min following treatment with PMA (200 nM, 20 min) in the nuclear and cytoplasmic protein extracts of MEF and PARP1-KO MEF. Representative results of 3 different experiments are displayed. (**B**) Right: A PARP1 dependent Erk activity in a cell-free system. Immunolabeled recombinant PARP1 (100 ng/sample) polyADP-ribosylated in the presence of NAD (10 µM), ATP (1 mM) and recombinant phosphorylated Erk2 (100 ng/sample). Recombinant Elk1 (600 ng/sample) was phosphorylated in this system by recombinant phosphorylated Erk2. PARP1 polyADP-ribosylation was attenuated in the presence of recombinant F domain of PARP1 carrying its Erk binding sites (aa556-1014 domain; 100 ng/sample), or in the presence of nicked DNA (ssDNA;1 mg/sample). The recombinant AB domain of PARP1 which includes its DNA binding sites (1–20 aa domain, 100 ng/sample) did not affect Erk-induced PARP1 activation. Representative results of 3 different experiments are displayed. Left: A ribbon structural model for the open conformation of PARP1 with consensus docking sites for phosphorylated Erk (orange spheres in blue shaded region) in its catalytic (CAT), helical (HD) and WGR domains (From: Figure 6A in ref #7).

In MEF, PMA induced Erk phosphorylation was accompanied by Erk translocation into the nucleus (Figure 1). Phosphorylated Erk was sampled in MEF nuclei and in their nuclear extracts (Figure 2) during 120 min after stimulation. A fast decay of phosphorylated Erk was measured in nuclei of PARP1-KO MEF (34), relative to the long-lasting Erk phosphorylation in nuclei of normal MEF (Figure 1). Erk phosphorylation decayed 90 min after treatment with PMA (200 nM, 15 min; Methods) in normal MEF, and 15 min after PMA-treatment in PARP1-KO MEF (Figure 1).

In addition, a low phosphophorylation of Erk substrates, transcription factors Elk1 and cFos was sampled in PARP1-KO MEF, in comparison to normal MEF (Figure 2). Erk phosphorylation in the cytoplasm of MEF was not affected by the genetic deletion of PARP1, indicating an exclusive effect of PARP1 on the activity of phosphorylated Erk in their nuclei (Figure 2). These results are in line with Erk-bound PARP1 activation and PARP1-dependent phosphorylated Erk activity promoting the expression of cfos (Figure 2A), which was previously observed in a cell free system (Figure 2B).

**Figure 3:**
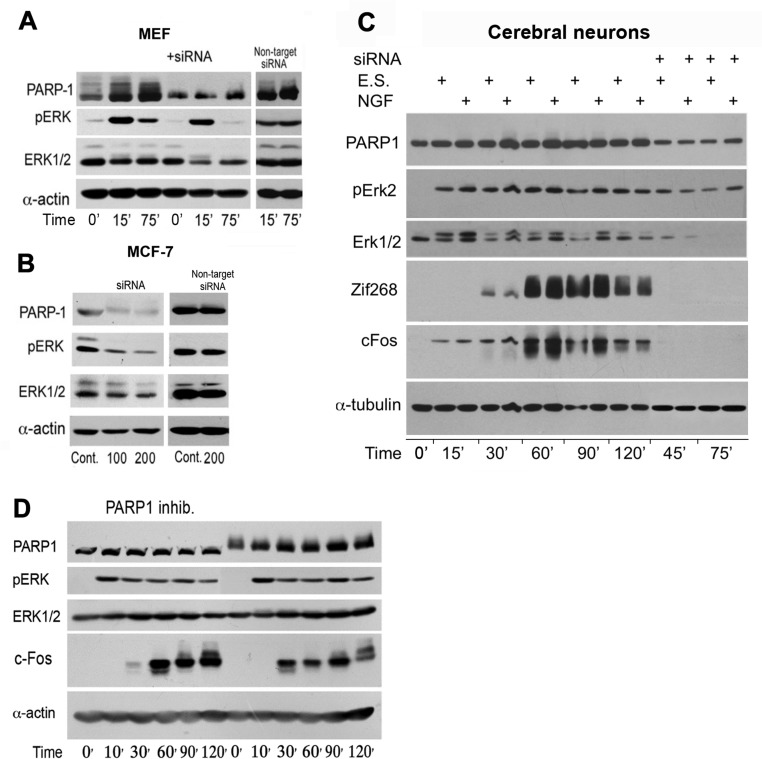
PARP1 mediates stimulation-induced Erk phosphorylation in the nucleus (**A**) PARP1 and phosphorylated Erk1/2 were monitored by immunolabeling during 120 min in MEF after stimulation with PMA (200 nM, 15 min). PARP1-silencing suppressed stimulation induced Erk phosphorylation in the nuclei of MEF and in nuclei of unstimulated human breast cancer MCF-7 cells (**B**). (**C**) PARP1, phosphorylated Erk1/2 and transcription factors c-Fos and Zif268 were monitored by immunelabeling during 120 min in cerebral cortical neurons after electrical stimulation (ES; 100 Hz, 1 sec, 3 repeats each followed by 10 sec pause), without or in the presence of nerve growth factor (NGF; 60 ng/ml, 5 min). Erk2 phosphorylation and proteins' zif 268 and c-Fos were hardly detected after PARP1 silencing. (**D**) PARP1 activity mediating Erk activity in MEF. A reversible inhibition of PARP1 in MEF treated with PJ34 (10 μM, 30 min) prior to PMA stimulation (200 nM, 20 min) prevented the following elevation of cFos (30 min after stimulation). Representative results of 3 different experiments are displayed for each of these experiments.

PARP1 silencing similarly suppressed a stimulation induced Elk phosphorylation in the nuclei of MEF stimulated by PMA, and human breast cancer cells MCF-7 (Figure 3A and 3B). Similarly, PARP1 silencing suppressed Erk induced IEG expression in stimulated cortical neurons (Figure 3C). PARP1 inhibitors although not interfering with PARP1 binding to phosphorylated Erk [5–7], could prevent chromatin relaxation due to polyADP-ribosylation of linker histone H1 [5–8]. In accordance, a transient PARP1 inhibition preceding stimulation with PMA, suppressed c-Fos synthesis sampled 30 min after stimulation (Figure 3D).

**Figure 4:**
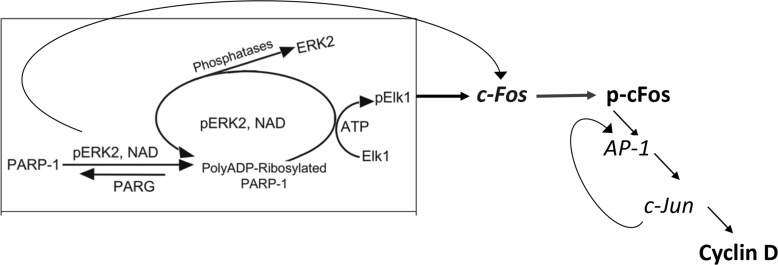
A flowchart presentation for PARP1 - Erk synergism controlling the expression of cyclin D

These results support the notion that PARP1 is required for preserving the activity of phosphorylated Erk in nuclei of stimulated MEF, and PARP1 activity mediates Erk-induced cFos synthesis promoting proliferation (Figure 4). This PARP1-Erk synergism could be a new target of treatments aiming to suppress proliferation [18].

## MATERIALS AND METHODS

### Antibodies and recombinant proteins

PARP1 and its recombinant domains were immunolabeled by a monoclonal antibody (Serotec, Cat # MCA1522; Oxford, UK), and a polyclonal antibody (Alexis, Cat # ALX210-302). ADP-ribose polymers (PAR) were immunolabeled (Alexis, cat #961004). Erk2 was immunolabeled by antibody directed against the c-terminal of Erk2 (#sc-154; Santa Cruz Biotechnology, CA, USA), phosphorylated Erk1/2, α-actin (Sigma), Elk1 and phosphorylated Elk1 and cFos were immunolabeled by antibodies purchased from Cell Signaling Technologies (MA, USA). First antibodies were labeled by fluorescent secondary antibody: CyTM2 (green) or fluorescent CyTM3 (red) conjugated affinity pure goat-anti-rabbit or goat anti-mouse secondary antibodies (Jackson ImmunoResearch). Recombinant PARP1 domains were prepared in the lab of Dr Francoise Dantzer (Strasbourg, France). Recombinant phosphorylated Erk2 was prepared in the lab of Prof Seger, as described before [5].

Recombinants Elk1 (Elk1fusion protein lacking the DNA-binding domain of Elk; Elk1 residues 307−428 coupled to GST; Cell Signaling Technologies). ssDNA was from Sigma (salmon sperm DNA carrying numerous single strand breaks) [5].

PARP1 activation is measured by [^32^P]polyADP-ribosylation of PARP1 in nuclear extracts of MEF incubated with [^32^P]NAD (1 μCi/sample; 1000 mCi/mmol; Amersham, UK) as described before [19]. It is also indicated by immunolabeling with antibodies directed against ADP-ribose polymers or antibodies directed against PARP1 [19].

Erk-induced PARP1 polyADP-ribosylation in a cell-free system has been described before [5, 6]. Recombinant PARP1 (100 ng/sample) was activated in the presence of [^32^P]NAD (1000 Ci/mmol; 1 mCi/sample; 20 nM) or unlabeled NAD, ATP (1 mM) and recombinant phosphorylated Erk2 (100 ng/sample). Recombinant Elk1 (600 ng/sample) was phosphorylated in this system by recombinant phosphorylated Erk2.

PARP1 silencing by siRNA has been described before [5]. Two sequences, aa800-807 and aa890-897, in the PARP1 catalytic domain were targeted for PARP1 silencing. PARP1 targeted siRNA was prepared by Darmacon (Lafayette CO, USA). For control we used a non-specific siRNA#2 (non-spec. rat siRNA; Darmacon). Cells in cell culture were transfected by XtremeGENE siRNA transfection reagent (Cat no. 04476093001, Roche Diagnostic, GmbH Mannheim, Germany). PARP1 silencing was achieved 72 hours after transfection with 100–200 nM siRNA.

Preparation of MEF from normal and PARP1 KO mice. PARP1(−/+) 129/Sv mice were donated by Dr Dantzer (Strasbourg) and bred for PARP1 (−/−) mice in Cohen-Armon’s lab (Tel-Aviv University) under the rules and regulations of the Institutional Animal Care and Use Committee. Mouse embryonic fibroblasts were prepared from normal and PARP-KO MEF according to a procedure developed in the lab of Dr. Dantzer [20].

Nuclear protein extracts were prepared according to a procedure described before [7, 21].

Treatment with PMA. MEF were treated with PMA (phorbol esther; 200 nM; 20 min) in serum deprived medium. PMA was then 5-times diluted by adding normal medium, and crude nuclei were prepared from MEF after various incubation periods with diluted PMA (40 nM).
